# Supplementary figures and images for: Predicting activatory and inhibitory drug–target interactions based on structural compound representations and genetically perturbed transcriptomes
Source: PLoS One. 2023 Apr 12;18(4):e0282042. doi: 10.1371/journal.pone.0282042 (PMC10096289; doi:10.1371/journal.pone.0282042)

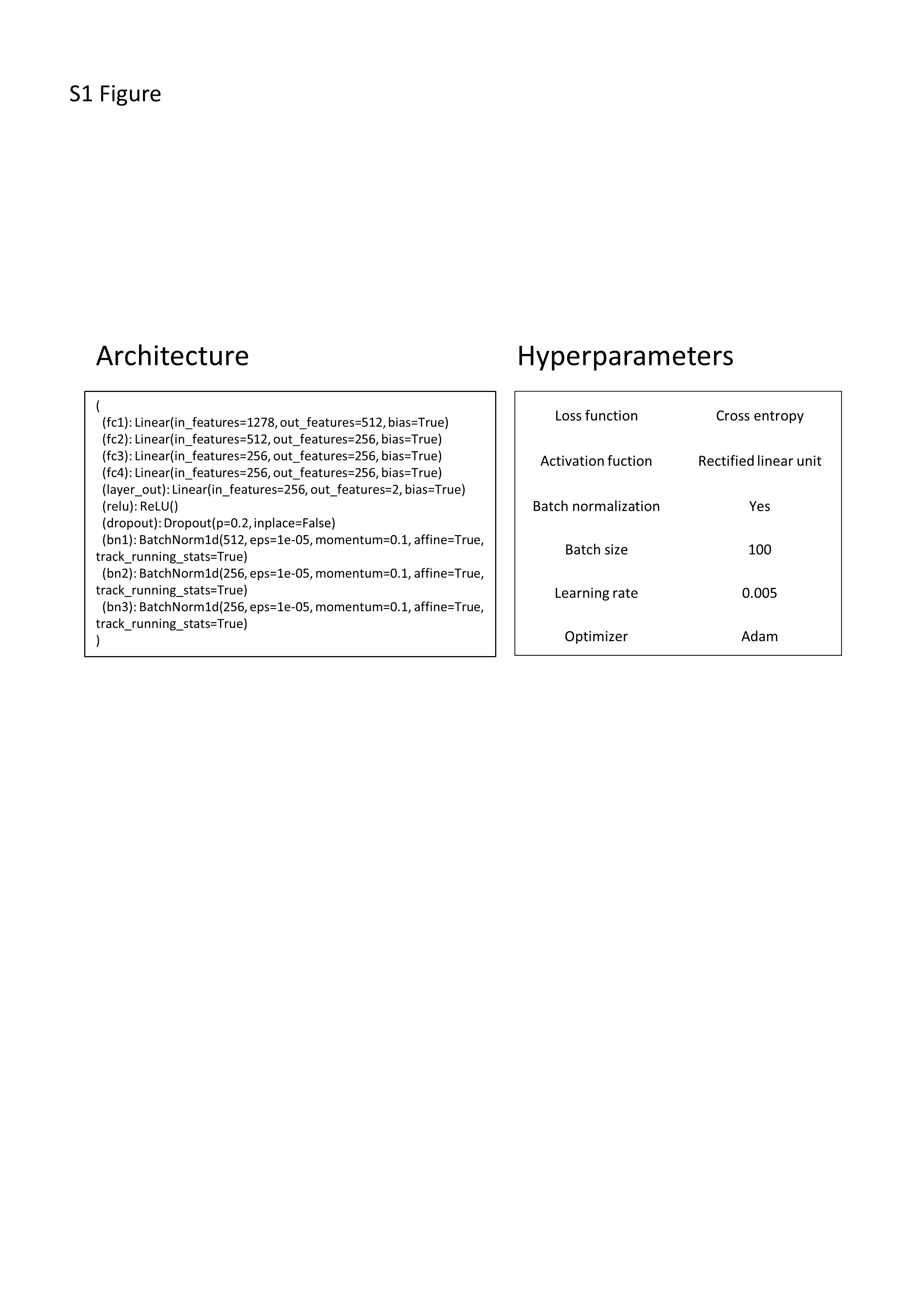

Supplement: S1 Fig — (TIF) [file pone.0282042.s006.TIF]

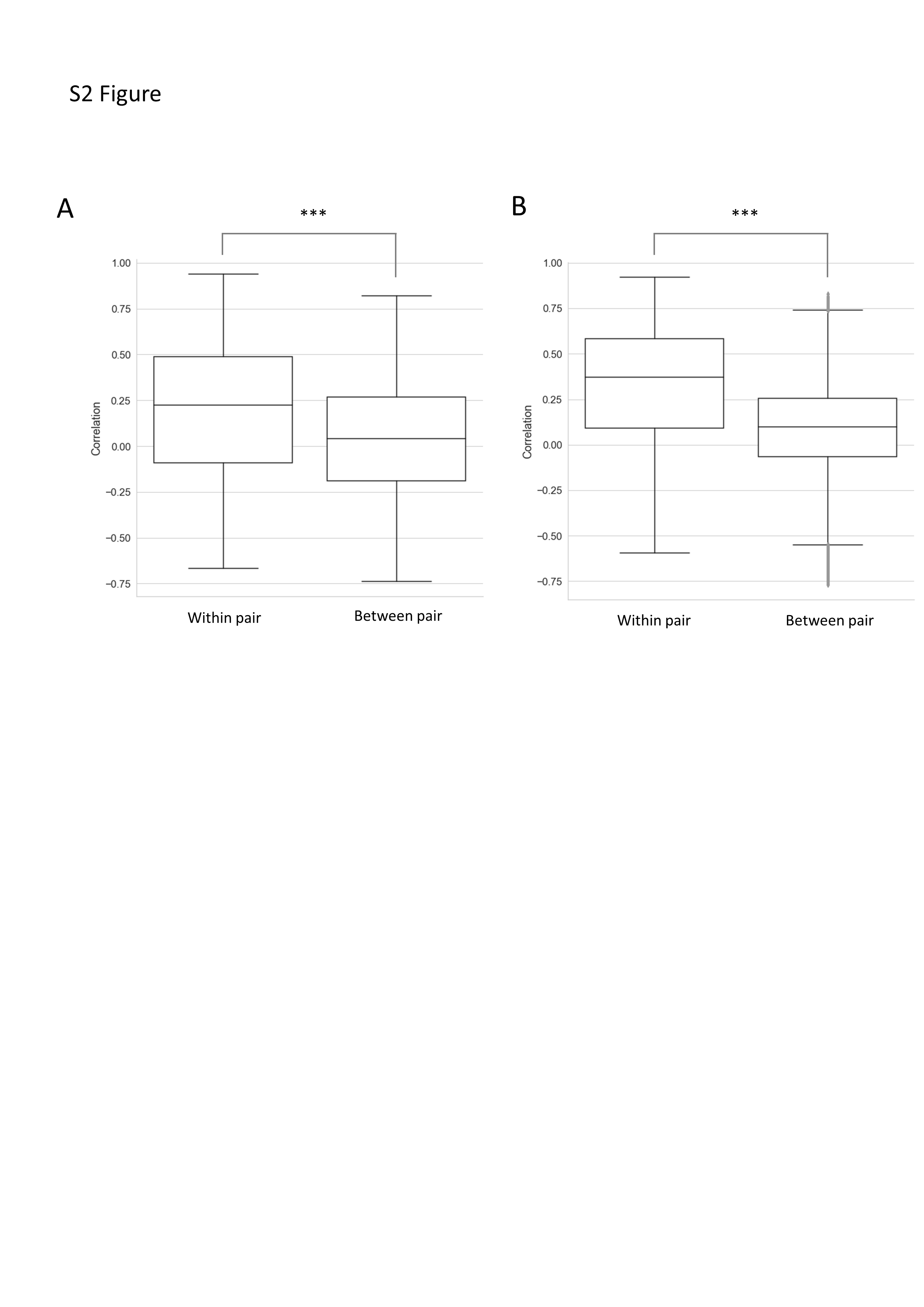

Supplement: S2 Fig — (TIF) [file pone.0282042.s007.TIF]

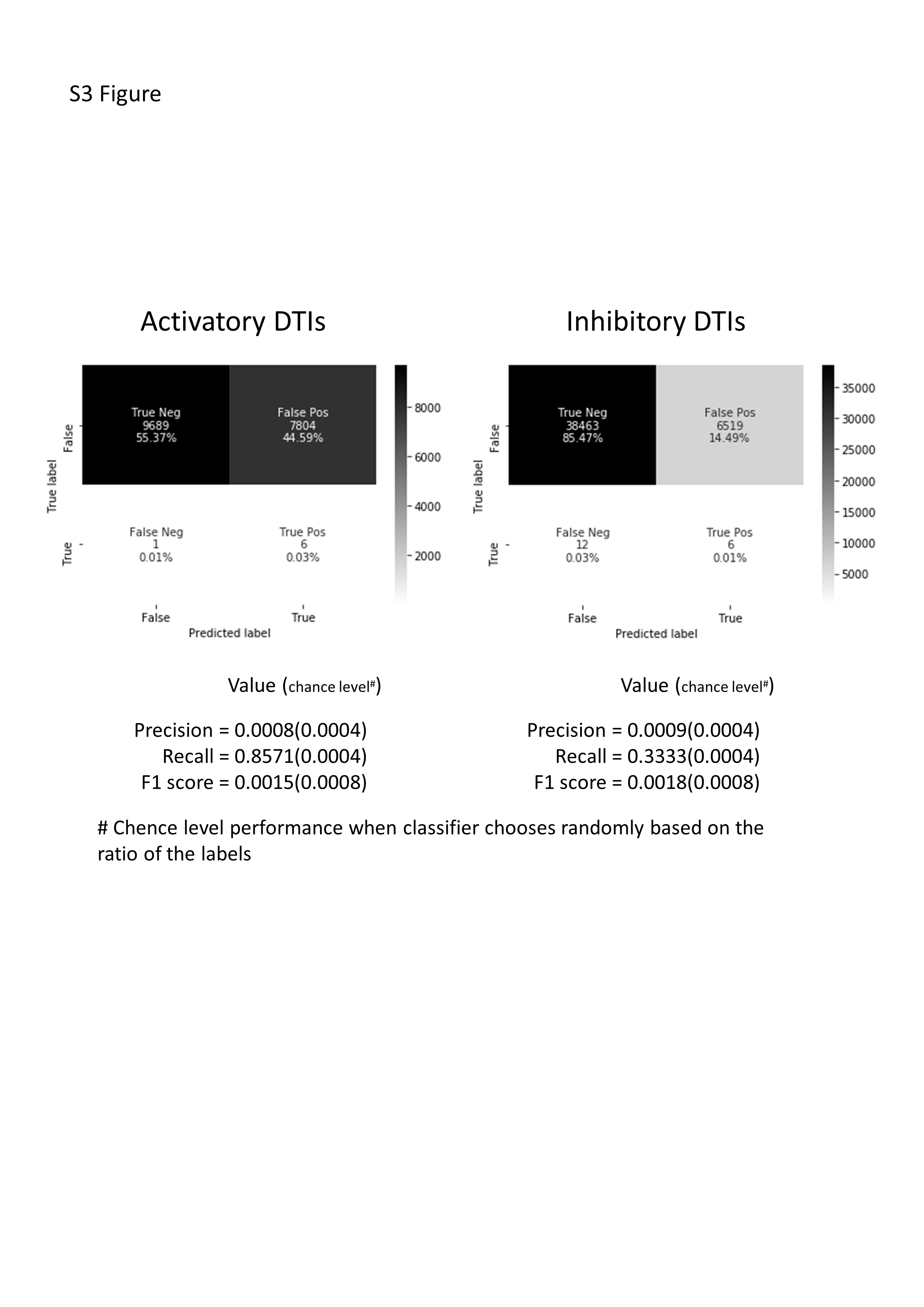

Supplement: S3 Fig — (TIF) [file pone.0282042.s008.TIF]
