# Supplementary material for: Predicting activatory and inhibitory drug–target interactions based on structural compound representations and genetically perturbed transcriptomes
Source: PLoS One. 2023 Apr 12;18(4):e0282042. doi: 10.1371/journal.pone.0282042 (PMC10096289; doi:10.1371/journal.pone.0282042)
Supplement: Attachment — Submitted filename: 220802_revision_comments.docx [file pone.0282042.s010.docx]

Reviewer #1:

In this manuscript, the authors presented a method to predict the activatory and inhibitory Drug-target Interactions. The structural compound representations (Mol2vec) and genetically perturbed transcriptomes (978 landmark genes) were used as the features for machine learning. The training datasets were collected from the Therapeutic Target Database 2.0 and DrugBank 5.1.7. The independent test sets were constructed from Drugbank and LIT-PCBA. Four machine learning methods were used for model training, and cascade deep forest has the best predictive performance. The topic is essential, but there are several major concerns that the authors need to address.

* The proposed method has only one prediction model for active DTIs. The predicted result for a DTI is either activatory or inhibitory. However, many relationships between drugs and targets are inactive. The application of the prediction model could be pretty limited to the availability of knowing interaction between drug and target.

We appreciate for the comments.

As the reviewers were concerned, the purpose of our model is not to classify the mode of action among known DTIs, but rather to predict whether DTI of interest is active or inactive with its mode of action (activated or inhibited). In addition, we found that AI-DTI could predict DTIs more accurately than previous models, and that the range of predictable compounds and targets was expanded. Therefore, the applicability of our model would not be limited to the availability of known interactions between drug and targets. Thanks to the comments, we found that the previous manuscript did not appropriately describe our aims of the model. To reduce potential confusion, we revised the build process and goals of AI-DTI to be clearer in the manuscript. (line 274-286).

* How many mol2vec-based compound features were used for a compound? Were they 300-dimensional embedding of Morgan substructures? How does the window size of 10 work?

As the reviewer mentioned, we used a mol2vec model that receives morgan substructure as input and converts it into a 300-dimensional vector. To describe window size of mol2vec, it would be useful to share the structure of mol2vec. Mol2vec is a NLP-inspired model for calculating compound embeddings. It is trained by calculating vectors that can best reconstruct the substructures contained in the Morgan fingerprint and its surrounding substructures. The window size of mol2vec is the number of surrounding substructures to be considered in the training phase.

* There are 1,278 features if the number of compound features is 300. The number of activatory DTIs in the original and additional are 457 and 887, respectively. It may cause the model to overfit if the number of features exceeds the number of positive/negative samples.

-> Thanks for pointing out this important issue.

As the reviewer points, the overfitting is a major issue in the application of machine learning models. The reviewer noted that this could be due to the ratio between the input function and the number of samples.

To minimize the overfitting issue, we considered various regularization terms such as max_depth, number of trees, and number of telerant rounds for cascade deep forest. Moreover, the trained model showed superior performance on various independent datasets, indicating that there were no significant overfitting issues in the trained model.

* The EFx%, precision, and true and false positive rate equations were wrong without parentheses.

Thank you for pointing our mistakes.

We add parentheses to the equation.

* Why is the number of inactive activatory DTIs (318,066) more than the number of compounds (130,412) multiply the number of targets (2) in the LIT-PCBA set?

Thank you for noticing our mistakes.

The number of target has been updated with correct information, which is the same as the information on the homepage (https://drugdesign.unistra.fr/LIT-PCBA/).

In lines 212-213, active and inactive DTIs should be described more clearly.

To reflect the reviewer's request, we describe in more detail considering active and inactive DTIs. (Line 201-204)

* In Table 4, the authors compared their method with the previous model (ref. 18). How to get the predictive performance of independent sets using the previous prediction model?

We appreciate for pointing out a critical issue.

Before revision, the performance of the previous model was derived from the supplementary tables in the previous study. It indicates that the performance of our model and the previous study were measured on different datasets. We realized that this was an unfair condition, so we constructed a common dataset and compared the performance with our model and the previous model. As a result of comprehensive comparative evaluation over hyperparameters of joint learning and classifiers of AI-DTI, we found that AI-DTI showed higher AUROC and AUPR than joint learning, even on the constructed dataset (Supplementary Table S2).

* In line 437, the authors indicated that approximately half of the DTIs (12/25) were successfully rediscovered. It is similar to random if the proposed model was for predicting the DTI is either activatory or inhibitory.

As we mentioned in reviewer’s comments 1, the chance ratio of DTI rediscovery is not 0.5 when considering the imbalance between active and inactive DTIs. To quantitatively support our opinion, we calculated a confusion matrix on the COVID-19 dataset and found that the F1-score was more than twice higher than the random chance level. Also, we found that that the prediction results of AI-DTI were significantly associated with known-unknown DTIs (p < 0.05, Fisher's Exact test and Chi-square test for activatory and inhibitory DTIs, respectively),

Reviewer #2: Reviewer Response:

The reviewer appreciates the efforts put forth by the authors. However, there are several concerns regarding the study that hinder its overall effectiveness.

Major:

* A fundamental issue with the submitted manuscript seems to be the combination of two types of features – chemical features and gene expression. The two types have different meanings in drug design. An inhibitor is usually designed to inhibit the activity of a target protein instead of changing the expression level of the target protein. For example, Gefitinib is an EGFR inhibitor, and the drug inhibits EGFR activity. When treated with the drug, the expression level of p-EGFR is inhibited, while the expression level of EGFR is similar. Therefore, combining the fingerprints of Gefitinib with the gene knock-down signature of EGFR may be not reasonable for use as features to predict the drug-target interaction.

-> Thanks for pointing out such an important point.

As the reviewers noted, when focusing on a single gene/protein, we agree with the reviewer’s comment that perturbations to protein functions and gene expression have different meaning in drug design. Specifically, when a drug inhibits protein, the expression level of protein does not change as you mentioned, but the inhibition of the protein can influence other gene expressions level and this response can be inferred by investigating genetic perturbed transcriptome. Using this assumption, several researchers have developed machine learning models to predict DTIs using the KO gene signature as target feature vectos of DTIs [Sawada et al., Xie et al., Lee et al.,]. The results of our study also indicate that DTIs can be accurately classified using the signature measured after gene perturbation, which supports that the genetic perturbation signature can be useful in predicting DTIs.

\# Reference

Sawada, Ryusuke, et al. "Predicting inhibitory and activatory drug targets by chemically and genetically perturbed transcriptome signatures." *Scientific reports* 8.1 (2018): 1-9.

Xie, Lingwei, et al. "Deep learning-based transcriptome data classification for drug-target interaction prediction." *BMC genomics* 19.7 (2018): 93-102.

Lee, Hanbi, and Wankyu Kim. "Comparison of target features for predicting drug-target interactions by deep neural network based on large-scale drug-induced transcriptome data." *Pharmaceutics* 11.8 (2019): 377.

* Regarding the feature generation, the use of mol2vec is interesting. However, concatenation of gene expression is a major flaw. We can use HDAC inhibitors as an example. Many HDAC inhibitors contain a hydroxamic moiety. The fingerprint for each compound would be different; however, this information is concatenated with additional information (known gene expression). Adding gene expression would then train a model to “assume” all compounds containing a hydroxamic moiety would induce the same gene expression. The information (mol2vec and the concatenated gene expression) would be better used separately.

-> Thanks for pointing out a potential issue with our model.

As the reviewer worried, the aim of concatenating a target and a compound vector is not to investigate the causality between them, but to provide an input feature for evaluating the reliability of DTIs. In the training process, the impact of the issues mentioned by the reviewer could be reduced through a data-driven manner. Let's consider the DTI dataset focused on HDAC as a same example. In the dataset, DTIs contains combinations of a compound containing a hydroxamic moiety and an HDAC-perturbed gene expression, and they are used to train a classification model. If the presence or absence of a hydroxam moiety does not significantly affect the discrimination of DTIs, then the trained model will not significantly consider feature vectors derived from the hydroxam moiety even when predicting DTI.

Also, the reviewer gives suggestion to use the target feature and the compound feature separately. There are *pros* and *cons* to using features separately and using them together. The approach that uses only compound information refers to the ligand-based approach, it shows high predictive performance when information on known ligands for a specific protein is sufficient. On the other hand, an approach with the concatenated use of target and compound feature vectors fall within a chemogenomic approach, and it can efficiently predict a wide range of DTIs. AI-DTI were proposed for predicting targets for natrual products or experimental small molecules for which evidence is lacking. Therefore, AI-DTI concatenated compound features and target features to take advantage of this advantage.

* In Figure 2, the authors show that they aggregated the transcriptome signatures from L1000 profiles. However, the information is aggregated across different cell lines. Following the workflow in Figure 2, it seems the authors combined the expression across different cancers (such as breast cancer and colorectal cancer cell lines). Combining these results does not seem reasonable.

-> As reviewers are concerned, drug-induced signatures in a single cell line are mixed effects of cell line-specific responses and changes in drug effects. Cell line-specific responses risk acting as noise in the prediction of DTI in the general context. Therefore, one way to reduce this risk is to compare the administration of the same drug to multiple cell lines and derive a common response data. To this end, by applying similarity-based weighted aggregation, we were able to obetain representative responses of drug administration while reducing cell-specific responses.

The authors reference models was developed by Sawada et al, which displayed information based on chemical treatment, gene knockdown, or gene overexpression. All three information by Sawada et al. is used as separated features. These steps seem more practical when compared to the authors’ submitted manuscript.

-> We appreciate for pointed out an important issue in terms of the practicality.

To address the issue, we performed a comprehensive comparison with the previous model. We first constructed a common dataset that can be used to compare performance without bias and compared the performance between our method and previous approaches. As a result of comprehensive comparative evaluation over hyperparameters of joint learning and classifiers of AI-DTI, we found that AI-DTI showed higher AUROC and AUPR than joint learning (Supplementary Table S2). In addition, the previous approach requires drug-induced signatures for the prediction. However, our method can predict DTIs for most compound with a 2D structure and a wider target. Taken together, we found that our model was more useful than the previous model in terms of prediction performance and predictable range of drugs and targets.

* Figure 4 is unclear. There are 2 sets of ROC and Precision-Recall Curve, but it is hard to determine which graph fits for predicting activity/inhibition by the authors in sections 3.4-3.5.

-> We appreciate for pointing out the ambiguity of the figure.

We added annotation which graphs are fitted for activatory and inhibitory DTIs.

Regarding Figure 4B, the AUC for AI-DTI (Mean = 0.61) is quite low. Are the compounds truly distinct from the training set?

-> There are three main reasons for the low AUC value evaluated in LIT-PCBA dataset. First, the performance was measured on an independent dataset. Second, the source of the dataset is heterogenous from the training set (expert-curated DTIs for training model vs. high-throughtput screening experiment in independent dataset). Finally, the problem was more difficult due to severe data imbalance. Nevertheless, our method showed superior performance compared to the conventional method, which supports the usefulness of our model.

We find that a small number of compounds contained in a known DTI appear as DTI for different targets in an independent dataset, but no exact matched DTIs for drug and target pairs. Therefore, we considered that the LIT-PCBA consists of an unseen DTIs in the training phase.

* A comparison between mol2vec and other popular chemical representations may be needed and would increase the study’s novelty.

-> Thank you for your suggestions to improve the novelty of the study.

According to the reviewer’s suggestion, we measured the prediction performance after changing the compound representation method to MACCS and morgan fingerprint methods and found that mol2vec still outperforms these methods (Table 3).

* The authors sought to test their model for COVID-19 DTI. However, insufficient explanation for why the analysis was performed or how it fits with the DTI study was given.

-> We appreciate for pointing out the potential ambiguity in our manuscript.

Our motivation for conducting a case study on COVID-19 was to evaluate whether AI-DTI could help the drug discovery process for an unseen disease. To clarify the goals of the analysis on COVID-19, we supplemented the rationale for why we performed a case study in section 3.6 (Line 435-439).

* Authors previously modified their manuscript to address reviewer concerns. However, these areas (in red) as well as the original contents contain grammatical issues or are not written clearly for readers.

-> To reflect the reviewer's comments, we comprehensively reviewed the manuscript and revise the manuscript. In particular, the last section of the introduction (line 79-84), the second paragraph of the overview of the results (line 274-284), and the performance evaluation with the existing model (line 319-349) have been comprehensively rewritten.

Minor:

* A confusion matrix illustrating the COVID-19 DTI results would be helpful.

-> We appreciate for the reviewer’s recommendation.

According to reviewer’s suggestion, we calculated a confusion matrix focused on DTIs between FDA-approved drugs and related targets with COVID-19, and found that AI-DTI achieved an F1-score more than twice the chance level (Supplementary Figure 3).

* Authors randomly selected their negative samples. The reviewer recommends using an additional database for negative samples (i.e. ChEMBL, PubChem, etc). Compounds can be separated based on reported bioactivity (IC50, Ki, or Kd) as a cutoff for negative samples.

-> Thanks for the reviewer's suggestion.

If sufficient number of experimentally validated negative samples are obtained, the reliability of the DTI prediction model can be improved.  Unfortunately, as reviewers knows, the number of verified negative DTIs samples is very insufficient. As an example, Souri et al obtained a negative sample of inactive DTIs from Chemble, but the number of them was only 2,057, which would be an insufficient number to train the classifiers.

\# Reference

Amiri Souri, E., et al. "Novel drug-target interactions via link prediction and network embedding." *BMC bioinformatics* 23.1 (2022): 1-16.

Another issue of using experimentally validated results as negative DTIs is the heterogeneity of target and drug types between positive and negative samples. It would induce a ‘shortcut learning’ issue, which the classifier will be trained to distinguish the types of drugs and targets appearing in each dataset, rather than evaluating the reliability of the DTI pair. On the other hands, random selection of negative DTIs from drug and target pairs included in the positive DTI data set can avoid this risk. Therefore, we selected negative samples by random selection of non-interacting pairs from these drugs and targets in each dataset.
