# Supplementary material for: Predicting activatory and inhibitory drug–target interactions based on structural compound representations and genetically perturbed transcriptomes
Source: PLoS One. 2023 Apr 12;18(4):e0282042. doi: 10.1371/journal.pone.0282042 (PMC10096289; doi:10.1371/journal.pone.0282042)
Supplement: Attachment — Submitted filename: 220912_re_revision_response to reviewer.docx [file pone.0282042.s011.docx]

Reviewer #1: Most comments have been addressed.

But the EFx% equation is still wrong without parentheses in numerator.

-> Thanks for pointing out our mistake.

We added parentheses to the EFx% equation.

===============

Reviewer #2: 1. The reviewer appreciates the authors' comments. While we agree that “inhibition of a protein can influence gene other expression levels”, the authors continue to assume that all compounds would result in the same gene expression level as the knock-down/overexpressed query target (i.e. same off-target hits, same protein-ligand binding mechanism, etc). As such, combining the compound fingerprints with knock-down/overexpression of genes as input features for establishing a model appears unreasonable.

The reviewer also appreciates the references given. Unfortunately, the referenced papers used transcriptome signatures as standalone features. Compared to the presented study, the referenced articles appear more reasonable for establishing a useful DTI network.

For example, Gefitinib, Erlotinib, and Lapatinib are EGFR inhibitors and the three compounds would result in different gene expressions. However, in the authors' model, the fingerprints of the three compounds will combine with the knock-down gene signature of EGFR. This assumes that the three compounds would result in the same gene expression level with the knock-down gene signature of EGFR. Therefore, the concatenation of gene expression and chemical information continues to be a major flaw to this study.

We agree with the reviewer’s opinion and believe that an approach without performance evaluations may be unreasonable or majorly flawed. To this end, we previously conducted various tasks to evaluate the efficiency of our model and found that our model outperformed the previous model and showed superior performance in various independent datasets.

Also, the reviewer expressed potential concerns on combining the target feature with the compound feature. There are *pros* and *cons* to using features separately and using them together. One of the advantages of using concatenated use of target and compound feature vectors is that it can efficiently predict a wide range of DTIs. In particular, AI-DTI can predict DTIs with mode of action for compounds for which only 2D structures are available because it only requires structure-based information of the compound and genetically perturbed transcriptome as input features. As a representative example, our previous study suggested that AI-DTI is an efficient method to identify candidate flavonoids for NAFLD, a representative liver disease [WY Lee et al., 2022]. Approaches cited by reviewer as being more efficient may be limited by their inability to predict the DTIs for these natural products, where the transcriptome is not measured. Taken together, we showed the reliability of the model through a comprehensive performance evaluation, which can resolve potential concerns for reasonability or flawless

\# Reference

- Lee, Won-Yung, et al. "Identifying candidate flavonoids for non-alcoholic fatty liver disease by network-based strategy." Frontiers in Pharmacology (2022): 1718.

2. The reviewer understands the authors’ attempts at reducing prediction noise. Unfortunately, aggregating the information across different cell lines is unreasonable in establishing an effective DTI. Aggregating this information could potentially produce transcriptional signatures that are no longer relevant to a given disease. As the goal of the authors appear to be identifying a drug’s target interactions, this would be problematic. As a result, aggregating information across cell lines would greatly impair the effectiveness of the given model.

-> As the reviewers are concerned, drug-induced transcripts in a single cell line may be biased towards the specific response of the measured cell line. Cell line-specific responses risk acting as noise in DTI predictions in the general context agnostic to specific diseases. Therefore, one way to reduce this risk is to derive common data by aggregating the responses of multiple cell lines administered the same drug. To this end, by applying similarity-based weighted aggregation, we were able to obtain representative responses of drug administration while reducing cell-specific responses.

3. Regarding the dataset, were the independent dataset randomly selected and removed from the training set? Was there an attempt at balancing the dataset and seeing how that would affect performance?

-> We appreciate the reviewer's comments.

The random selection process is used in the classifier training phase to obtain negative samples, but not in the generation of independent datasets. In the independent dataset, all unknown drug-target interaction pairs were assigned as negative samples. Therefore, we considered the reviewer's comment as the following three points: 1) whether to balance at the training stage, 2) risk of data leakage on independent datasets, and 3) whether balances on independent datasets are possible

Data balancing is a pivotal factor that significantly affects prediction performance during classifier training. A previous study showed that balancing positive and negative samples is an efficient way to improve the performance of classifiers in predicting DTIs [Sawada et al.,]. Similarly, we found superior performance when the ratio of positive: negative samples was set to 1:1 than when the ratio was 1:10 (Result are not shown).

The reviewer mentioned the risk of data leakage on independent datasets. Confirming that the independent dataset consists of an unseen dataset is an important issue in evaluating the generalized ability of a model. To this end, we compiled DTIs from other sources such as Drugbank, and LIT-PCBA to an independent dataset. We removed the DTIs that overlapped with the training dataset so that the independent dataset consisted of only unseen samples.

Finally, reviewers commented the balancing on independent datasets. Random selection of negative samples is also possible in independent datasets, but the usefulness of this method should be reconsidered given the actual drug development environment. DTIs are imbalanced problems with significantly fewer positive samples than negative samples. If an independent dataset is processed into a balanced dataset, there is a greater risk that the performance in the dataset will not match the actual application situation. Therefore, an evaluation approach on independent data without data balancing may be a more efficient way to evaluate performance in practical applications of drug discovery.

\# Reference

- Sawada, Ryusuke, et al. "Predicting inhibitory and activatory drug targets by chemically and genetically perturbed transcriptome signatures." *Scientific reports* 8.1 (2018): 1-9.
