# Supplementary material for: Predicting activatory and inhibitory drug–target interactions based on structural compound representations and genetically perturbed transcriptomes
Source: PLoS One. 2023 Apr 12;18(4):e0282042. doi: 10.1371/journal.pone.0282042 (PMC10096289; doi:10.1371/journal.pone.0282042)
Supplement: Attachment — Submitted filename: 221128_R3_revision_response.docx [file pone.0282042.s012.docx]

1. The authors concatenated gene expression with chemical information, with the assumption that all compounds would produce with the same gene expression level (same off-target hits, protein-ligand binding mechanism, etc). In the example given previously, Gefitinib, Erlotinib, and Lapatinib are EGFR inhibitors, but as reported previously, can produce different gene expression profiles in cell lines (We et al., 2020). As such, combining the compound fingerprints with knock-down/overexpression of genes, with the assumption that they have the same expression profile, as input features for establishing a model appears unreasonable.

Wei, Nan, et al. “transcriptome profiling of acquired gefitinib resistant lung cancer cells reveals dramatically changed transcription programs and new treatment targets.” Frontiers in Oncology (2020):

-> Thank you for the potential practical issue. To reflect reviewer’s concern, we conducted a case study focusing on EGFR inhibitors suggested by the reviewer.

Although gefitinib, erlotinib, and lapatinib are all EGFR inhibitors, each drug has different target information (Figure A. The figure is attached to the file uploaded to the reviewer's response file). For example, lapatinib has additional targets such as HER2 and eEK-2K, and it can be expected that it would have different properties from signatures measured after EGFR silencing. On the other hand, in the case of gefitinib and erlotinib, which relatively have few or no other targets, it can be expected that they are relatively similar to the signatures measured after EGFR silencing.

To evaluate those expectation, we systematically measured the correlation between signatures of the EGFR inhibitor and EGFR silencing Using CMap data (GEO number: GSE92742), which contains 205,034 drug-induced signatures. We found that the signatures measured after administration of erlotinib and gefitinib were higher than lapatinib. Interestingly, the signatures of gefitinib and erlotinib calculated by applying the aggregation method showed correlation values of 0.538 and 0.68, respectively (Figure B).

To check the significance of the observed result, we calculated the relative ranking of the correlation value for signatures measured after EGFR silencing. Relative ranks were calculated by comparing the drug's correlation value with the drug-perturbed signature (n=205,034) included throughout the GSE dataset. The result showed that the relative ranking of aggregated signature of gefitinib and erlotinib were 0.995 and 1 percentiles, which support the similarity between genetic perturbed signatures and drug-induced signatures (Figure C).

On the other hands, we found that the correlation value and its relative ranking of lapatinib with data aggregation was only -0.13 and 0.21, respectively. This indicates that drugs with multi targets are not suitable for embedding information of a single target. Rather, the genetically perturbed transcriptome, which adjusted for the off-target bias, would be more suitable for embedding information of a specific target. The discrepancies in the expression profiles of EGFR inhibitors in the papers cited by the reviewers highlight the multi-target nature of the drugs and support that drug-induced transcriptomes are not an optimal method for target embedding.

2. The reviewer appreciates the authors explanation of the reducing prediction noise in the submitted manuscript. Unfortunately, it does not assuage issues with response bias. Again, aggregation of information across cell lines could potentially produce transcriptional signatures that are no longer relevant to a given disease. Because the authors goal is to identify a drug’s target interactions, this continues to be problematic.

The work presented by the authors, aggregating and then giving weight to responses across different cell lines would require additional analysis (such as studying an example drug and its associated pathways) to prove the effectiveness of the model.

-> As shown in the case study above, we show that the aggregation method is the efficient way to obtain generalized vector embedding. Also, we found that the aggregation method can broaden the predictable number of targets. We counted the number of targets for which the genetic perturbed transcriptome was measured for each cell line. The result showed that the number of targets that can be additionally predicted through the aggregation method increases by at least 80 and 11 (approximately 15%) for activatory and inhibitory targets. To reflect our finding, we revised the characteristics of the aggregation method in the manuscript line 131-133.

Figure. Case study for EGFR inhibitor-induced signatures and signature measured after EGFR siliencing

A. EGFR-inhibitors and its target information. Target information were retrieved from TTD (accessed on november 28 2022)

B. Correlation distribution between EGFR inhibitor-induced signatures and aggregated EGFR inhibition signatures. Arrow points indicate spearman correlation of aggregated signatures for each drug. Others refers remaining all drugs in the GSE dataset except 3 EGFR inhibitors.

C. Relative ranking between EGFR inhibitor-induced signatures and EGFR inhibition signatures. A higher rank indicates higher correlation with EGFR siliencing than other signatures. The stars in each boxplot indicate the relative rank of the aggregated drug signatures.

D. Distribution of targets whose genetically perturbed transcriptome was measured by cell line
