# Supplementary material for: Predicting activatory and inhibitory drug–target interactions based on structural compound representations and genetically perturbed transcriptomes
Source: PLoS One. 2023 Apr 12;18(4):e0282042. doi: 10.1371/journal.pone.0282042 (PMC10096289; doi:10.1371/journal.pone.0282042)
Supplement: Attachment — Submitted filename: 230113_R4_comment response.docx [file pone.0282042.s013.docx]

The reviewer appreciates the efforts put forth by Lee et. al. However, major concerns remain regarding their submitted manuscript.

1. Chiefly, the authors concatenated gene expression with chemical information, with the assumption that all compounds would produce the same expression level. Concatenating gene expression with chemical information would present inaccurate results. This is due to compounds producing different gene expression levels. The authors give kinase inhibitors as examples. While Gefitinib and Erlotinib are selective EGFR inhibitors, both inhibitors also exhibit a number of off-targets, with large-scale screening available on the Guide to Pharmacology website.

As the authors mentioned, their results indicate that “drugs with multi targets are not suitable for embedding information of a single target.” The selectivity profile for these inhibitors in their example, Gefitinib and Erlotinib have different off-target inhibitory patterns throughout the human kinome. As a result, it would not seem reasonable to concat knock-down/overexpression of genes with chemical information.

-> Thank you for the valuable feedback provided by the reviewer. We appreciate the effort put forth in reviewing our manuscript.

In regards to the concern about concatenating gene expression with chemical information, we understand the reviewer's opinion that different compounds may produce different gene expression levels. However, our approach utilizes an aggregation method that aims to control for non-biological noise in transcripts, as inspired from the method used by Subramanian et al. Additionally, unlike traditional biological data, machine learning essentially needs a representative vector for a specific entity, which is an essential step in the machine learning approach. Studies such as Fernández-Torras et al. have shown that this embedding method for drugs can effectively characterize the drug itself and other drugs with similar performance to the drug-treatment transcriptome.

Furthermore, the reviewer's concern that concatenating gene expression with chemical information would present inaccurate results is not supported by current research in the field. Methods for calculating embedding for targets and compounds using gene expression and chemical fingerprinting are already widely used in the prediction of drug-target interactions, as shown in the studies by Lim et al. and Bagherian et al. Our study has already demonstrated that combining these methods can accurately predict activatory and inhibitory drug-target interactions for most compounds. Therefore, we believe that this point should be understood as a contribution to the novelty of our study, not an issue of accuracy.

In regards to the concern about off-target effects, our approach takes into account these limitations by employing an alternative strategy that uses a genetic perturbed transcriptome as an embedding for the target. This approach aims to ensure that the results are specific to the target, rather than being influenced by off-target effects. Our experiments focusing on EGFR inhibition in previous rounds have shown that this method is more efficient in characterizing specific target information compared to using drug-treated transcriptome. We understand the reviewer’s previous concern, but we believe that our approach has been validated by the experiments we performed and that the concerns brought up by the reviewer were already addressed in previous rounds of review.

\# References

Subramanian, Aravind, et al. "A next generation connectivity map: L1000 platform and the first 1,000,000 profiles." Cell 171.6 (2017): 1437-1452.

Fernández-Torras, Adrià, et al. "Integrating and formatting biomedical data as pre-calculated knowledge graph embeddings in the Bioteque." Nature Communications 13.1 (2022): 1-18.

Lim, Sangsoo, et al. "A review on compound-protein interaction prediction methods: data, format, representation and model." Computational and Structural Biotechnology Journal 19 (2021): 1541-1556.

Bagherian, Maryam, et al. "Machine learning approaches and databases for prediction of drug–target interaction: a survey paper." Briefings in bioinformatics 22.1 (2021): 247-269.

2. We appreciate the effort put forth by the authors. However, there are concerns that are continued to not be adequately addressed. Again, aggregation of information across cell lines could potentially produce transcriptional signatures that are no longer relevant to a given disease. Concatenation of gene expression with chemical information would not assuage these concerns. Because the authors’ goal is to identify drug target interactions, this continues to be problematic.

-> We appreciate the feedback provided by the reviewer and understand their concerns.

However, we would like to clarify that the transcriptional signatures used in this study, specifically the genetically perturbed transcriptome, are not associated with any specific disease. The purpose of our study is to develop a machine-learning model that can predict drug-target interactions, and the aggregation method is used to compute representative vector embeddings for more diverse targets. Although our model is not specialized for any specific disease, we would like to highlight that our model has been successfully applied to various diseases, as demonstrated in case studies of COVID-19 and in our previous study on non-alcoholic fatty liver disease [Lee et al.].

Furthermore, we would like to state that it is important to avoid mentioning potential issues that did not appear in our study and as such, we have taken care to not present any concerns that were not addressed in the paper. We are open to making any further revisions and adjustments that are deemed necessary to ensure that our study meets the standards of academic research.

\# Reference

Lee, Won-Yung, et al. "Identifying candidate flavonoids for non-alcoholic fatty liver disease by network-based strategy." Frontiers in Pharmacology (2022): 1718.
